# Bergamot and Olive Extracts as Beer Ingredients: Impact on Cell Viability, Reactive Oxygen Species, and RNA Expression of Antioxidant Enzymes

**DOI:** 10.3390/foods14122012

**Published:** 2025-06-06

**Authors:** Maria Laura Matrella, Bruna Amenta, Francesco Canino, Angela Maffia, Tiziana Cocco, Mariateresa Russo, Adele Muscolo

**Affiliations:** 1 Department of Translational Biomedicine and Neuroscience “DiBraiN”, Biochemistry Section, University of Bari “Aldo Moro”, 70124 Bari, Italy; maria.matrella@uniba.it (M.L.M.); bruna.amenta@uniba.it (B.A.); 2 Department of AGRARIA, Mediterranea University, Feo di Vito, 89122 Reggio Calabria, Italy; francesco.canino@unirc.it (F.C.); angela.maffia@unirc.it (A.M.); mariateresa.russo@unirc.it (M.R.)

**Keywords:** functional craft beer, antioxidant properties, bergamot juice, olive extract, oxidative stress

## Abstract

This study explores the incorporation of bergamot juice and olive extract as functional ingredients in craft beer and their effects on antioxidant activity and cellular oxidative stress. Lyophilized beer samples were applied to human fibroblast cells at concentrations of 0.31 and 0.62 µg/µL for 24 and 48 h. Cell viability, reactive oxygen species (ROS) levels, and antioxidant gene expression were evaluated. Beers enriched with bergamot (Heraclea) and olive extract (Elais) significantly reduced ROS levels compared to base beers (Blanche and Weiss), particularly at lower concentrations and shorter exposure times. However, prolonged treatment showed variable effects, suggesting possible concentration- and time-dependent pro-oxidant behavior. Gene expression analysis revealed the upregulation of the antioxidant enzyme SOD2 in all samples except Elais under oxidative stress, indicating differential cellular responses. These findings suggest that functional beers enriched with plant extracts may offer antioxidant benefits and support cellular defense mechanisms, representing a promising direction in wellness-oriented brewing.

## 1. Introduction

Beer, one of the oldest and most widely consumed alcoholic beverages globally, has evolved significantly in terms of ingredients and production methods. Historically, beer was crafted using simple ingredients: water, malted barley, hops, and yeast. In recent years, health concerns and a general desire for a healthier lifestyle have led to an increased demand for functional beers, and craft beer production has flourished with an increased emphasis on innovation and experimentation [1]. Modern brewers have explored the use of various botanicals and extracts to enhance the flavor, aroma, and content of bioactive compounds with functional properties in beer, creating new sensory experiences for consumers [2,3]. Among these innovations, incorporating plant-based extracts has received considerable attention due to their potential to enrich beer with unique sensory profiles such as bergamot (*Citrus bergamia*) and olive (*Olea europaea*) [4]. Bergamot, a citrus fruit native to the coastal regions of southern Italy, is best known for its distinctive fragrance. In the past, bergamot was mainly harvested for its essential oil, while by-products such as the juice, pulp, and seeds were considered industrial waste. However, in recent years, a growing body of research has highlighted the significant nutraceutical potential of these overlooked parts of the fruit [5]. Studies have demonstrated that bergamot when consumed fresh or as a juice, offers an impressive array of bioactive compounds that contribute to its health benefits. Unlike other fruits of the same genus, bergamot is particularly rich in a diverse range of phytochemicals and nutraceuticals, including organic acids, limonoids, phenolic acids, and flavonoids. These compounds have been shown to exhibit antioxidant, anti-inflammatory, and antimicrobial properties, suggesting that bergamot may offer various therapeutic benefits when incorporated into human diets [6,7,8]. Key compounds such as bergapten, flavonoids (e.g., rutin, quercetin), and coumarins have been shown to exhibit significant biological activities, including the modulation of oxidative stress and the inhibition of pro-inflammatory pathways. These bioactive compounds are thought to provide numerous health benefits, including protecting cells from oxidative damage, support for cardiovascular health, and potential anticancer effects [9]. The addition of bergamot extract into beer could offer a novel way of combining the antioxidant potential of the fruit with the functional properties of beer, promoting health-conscious consumption. Beer has a flavor profile that depends not only on its core ingredients—water, malted barley, hops, and yeast—but also on a variety of adjuncts and additives. In modern brewing, especially within the craft beer movement, brewers frequently experiment with additional substances to diversify flavor, aroma, and mouthfeel. These additives can enhance traditional styles or create entirely new taste experiences, broadening the sensory appeal of beer. One widely used additive is fruit, which imparts natural sweetness, acidity, and complex aromas. For example, adding raspberries or cherries during fermentation enriches the beer with tartness and vibrant red hues, common in styles like fruited lambics. Besides flavor, fruits contribute bioactive compounds such as polyphenols and anthocyanins, known for their antioxidant and anti-inflammatory properties, which may offer some protective cardiovascular benefits when consumed in moderation [10]. Honey serves as both a fermentable sugar source and a flavoring agent, imparting subtle floral notes and a light sweetness. Beyond taste, honey is rich in bioactive compounds like phenolic acids and flavonoids, which exhibit antimicrobial and antioxidant properties [11]. These may support gut health and immune function, though the brewing process can reduce the potency of these compounds to some extent. In darker beer styles, coffee and cocoa nibs are often used to infuse deep roasted flavors and aromas. These additives bring along bioactives such as caffeine, theobromine, and polyphenols. Caffeine offers mild stimulant effects, while cocoa polyphenols are linked to cardiovascular health benefits and antioxidant activity, adding a potential functional dimension to the beer [11]. Finally, wood-aging—employing oak barrels or chips—introduces flavors like vanilla, coconut, and smokiness due to compounds like lactones, tannins, and vanillin. Some of these compounds also have antioxidant effects, and tannins in particular can contribute to the beer’s astringency and mouthfeel, as well as having mild antimicrobial properties [12].

The bio compounds and the essential oils from bergamot, cultivated in the province of Reggio Calabria, are of exceptional quality compared to those derived from bergamot cultivated outside this area. The superior quality of Reggio Calabria’s bergamot is largely attributed to the unique microclimate and soil conditions, which provide an ideal growing environment and play a critical role in determining its distinctive qualitative characteristics [13]. Recent studies have confirmed that these environmental factors are key in influencing the chemical composition and therapeutic potential of bergamot. Similarly, olive extract, derived from the fruit of the olive tree, is well-documented for its wealth of polyphenolic compounds such as oleuropein, hydroxytyrosol, and oleocanthal [14,15]. These compounds have potent antioxidant, anti-inflammatory, and antimicrobial properties that have been associated with a variety of health benefits, particularly in the context of the Mediterranean diet. Olive extracts are believed to support heart health by improving lipid profiles, reducing blood pressure, and protecting against oxidative stress, while also exhibiting antimicrobial properties that could enhance the safety and preservation of foods and beverages [16]. The addition of olive extract to beer may not only enhance its organoleptic characteristics but also contribute to its functional properties by providing these bioactive benefits. As reported by De Bruno et al. [17], the olive of the cultivar *Carolea* contained more total phenolic compounds and had higher antioxidant activity expressed as DPPH than other Calabrian cultivars. The superior quality of the cultivar Carolea can be attributed to the ideal growing environment that plays a critical role in determining its distinctive qualitative characteristics [17].

This manuscript aims to explore the use of bergamot juice extracts and olive extracts as functional ingredients in craft beer production. We will investigate their impact on key biochemical processes including cell viability, reactive oxygen species (ROS) levels, and the regulation of RNA expression related to antioxidant enzymes. By evaluating the effects of these extracts on these molecular pathways, we aim to understand how these botanicals can potentially enhance the health-related qualities of beer, making it not just a beverage but a functional food that supports wellness. Furthermore, we will discuss how the integration of such extracts in brewing can shape the future of craft beer, providing an innovative and health-conscious option for consumers seeking both flavor and potential health benefits. 

## 2. Materials and Methods

### 2.1. Sample Preparation

In this study, we analyzed and compared the chemical and sensory properties of two craft beers produced in Calabria: Heraclea Blanche, enriched with unfiltered and unpasteurized bergamot juice extract, and Elais Weiss, which contained unfiltered and unpasteurized olive extract. Both Calabrian craft beers were compared to their respective base varieties (Blanche and Weiss), which were free of any added extracts, to assess how the natural additions affected the aromatic profiles, flavor characteristics, and functional properties.

The beers have been produced in EPICA Brewery (Messina, Sicily, Italy). To ensure consistency in the production process, we utilized a 20-hectolitre Tanker EVO 2000 BBC Inox brewing system, designed to optimize output without compromising quality. This modern, fully automated brewhouse skillfully integrates advanced technology with the adaptability typical of artisanal brewing. Automation enables precise replication of established recipes, improving both production efficiency and energy use, while allowing brewers to fully highlight the character of the raw ingredients. Every stage has been meticulously engineered to minimize oxygen exposure from the milling of the malt to the transfer into fermentation tanks. Additionally, the entire system is equipped for automatic cleaning and sanitization. The brewing setup also includes a cold chain system, which is essential for preserving the product’s quality during the stabilization phase. For packaging, the brewery operates an automatic isobaric bottling line under nitrogen (Isobaric, Gai), which is crucial for minimizing oxidation risks.

For the Blanche beer, a carefully balanced mixture of malt, barley, and oats was used, with Styrian Golding and Czech Saaz hops providing aroma and bitterness. An extract of bergamot juice, processed to remove terpenes, was added to enhance the flavor. The Weiss beer was produced using malted barley and Hallertau Hersbrucker hops, with an addition of olive fruit extract from the Carolea cultivar to impart distinctive notes. Specifics regarding the production parameters, ingredient concentrations, and mixing ratios are proprietary. Each beer type underwent fermentation in triplicate to ensure reproducibility.

Each sample was analyzed in triplicate for each batch and beer variety. Before testing, the samples were degassed at 20 °C using a magnetic stirrer to ensure the complete release of dissolved gases, thus standardizing the conditions for sensory and chemical analysis, as reported in Muscolo et al. [4]. This procedure allowed for a more accurate evaluation of the changes in flavor, aroma, and functional properties induced by the addition of bergamot and olive extracts.

### 2.2. Determination of Antioxidant Compounds

#### 2.2.1. Total Phenolic Content (TP)

Degassed and filtered (0.45 µm membrane) beer samples were diluted (1:5) with deionized water and the total phenolic content (TP) was determined according to Singleton & Rossi [18]. Absorbance was measured at 725 nm, and the concentration of TP was quantified using a tannic acid standard curve (0–500 mg/L). The results were expressed as milligrams of tannic acid (TA) per liter of extract.

#### 2.2.2. Total Flavonoid Content (TF)

Degassed and filtered (0.45 µm membrane) beer samples were diluted in methanol (1:5) and were used to detect total flavonoid content (TF) using a colorimetric aluminum chloride (AlCl_3_) method based on the procedure by Chang et al. [19]. Flavonoids react with aluminum chloride to form a flavonoid -Al^3+^ complex, which was then detected at 510 nm. The intensity of the resulting yellow color is proportional to the total flavonoid concentration in the sample. A quercetin standard curve (0 to 100 mg/L) in methanol was used. The results were expressed as milligrams of quercetin (QE) per liter of extract. 

#### 2.2.3. Total Carbohydrates and Ascorbic Acid Detection

Total carbohydrates were determined by the anthrone method with minor modifications [20]. Sugars react with anthrone reagent under acidic conditions, resulting in a blue-green color. After mixing the samples with sulphuric acid and anthrone reagent, the solution was boiled, cooled, and the absorbance was measured at 620 nm. A calibration curve was prepared using known concentrations of glucose to quantify the sugar content. This method quantifies both reducing and non-reducing sugars. For ascorbic acid determination, the protocol described by Muscolo et al. [20] was followed. 

#### 2.2.4. Polyphenolic Profile Determination

Beer samples were degassed by magnetic stirring (500 rpm) for 8 h and filtered through a 0.45 μm regenerated cellulose filter (Aisino Corporation). The polyphenolic profile was analyzed using ultra-high-performance liquid chromatography (UHPLC) with a Photo-Diode Array (PDA) detector (Shimadzu, Milan, Italy). The UHPLC system was equipped with a column oven (CTO-20AC), an autosampler (SIL-30AC), and an in-line degasser (DGU-20A5R). The chromatographic separation was performed on a Kinetex C18 column (50 mm × 3 mm × 1.7 μm particle size; Phenomenex), with a Kinetex C18 guard column (Phenomenex) used to protect the analytical column [21]. 

The optimized chromatographic conditions were as follows: mobile phase A was water with 0.1% formic acid, and mobile phase B was acetonitrile with 0.1% formic acid. The flow rate was set at 0.6 mL/min, and the column temperature was maintained at 40 °C. Gradient elution was performed: 1% B for 5 min, followed by a 15 min gradient from 1% to 30% B, and then a 7.5 min gradient from 30% to 65% B, before washing and reconditioning the system. The total separation time was approximately 28 min. 

The PDA detector was set with a spectrum resolution of 256 nm, a split width of 8 nm, and a sampling rate of 40 Hz. Data acquisition was performed over a wavelength range of 190–400 nm, and chromatograms were recorded at the optimal absorbance for the compounds of interest [21]. 

#### 2.2.5. Validation Method for Polyphenols

Seven concentration levels of the polyphenolic standards were prepared with methanol from a 1000 mg/L stock solution with concentration range of 0.5–120 mg/L. Five analyses were performed for each concentration level with the HPLC-PDA system under optimized chromatographic conditions. Retention time, instrumental recovery, and percentage relative standard deviation (RSD%) were determined using the fourth level (*n* = 4) of each calibration curve (Table 1).

### 2.3. Lyophilized Sample Preparation for Cell Culture Treatments

The beers have been lyophilized using a LABCONCO instrument (−0.133 mBars, −50 °C) purchased by Analytical Control De Mori s.r.l., Milano Italy. Lyophilization is a technique employed in beer processing to remove ethanol and stabilize bioactive compounds, thereby preserving sensory attributes and facilitating the creation of powdered beer products. Lyophilization involved three primary stages: (1) beer samples were cooled to sub-zero temperatures, typically around −55 °C, to solidify the water content; (2) under reduced pressure (approximately 0.2 mbar), the frozen water sublimated directly from ice to vapor, removing the majority of water content; (3) the remaining bound water molecules were removed by gradually increasing the temperature, ensuring the final product had minimal residual moisture. This process effectively removed ethanol and water while preserving heat-sensitive compounds, making it suitable for the production of stable beer powders. The duration of the process was (3 h) with a balanced removal of ethanol and water, retaining more volatile compounds.

The lyophilized beer samples were dissolved in phosphate-buffered saline (PBS), filtered through a 0.22 µm membrane filter and sterilized. The final concentration of the stock solution was 33.4 mg/mL for Blanche basal beer (B) and Blanche basal beer with the addition of the bergamot extract Heraclea (H) and 53.82 mg/mL for Weiss basal beer (W) and Weiss basal beer with the addition of the olive extract Elais (E). For the cell culture treatments, the concentrations of the beer samples tested were 0.31 and 0.62 µg/µL.

### 2.4. Cells and Culture Conditions

Primary fibroblasts from a healthy subject obtained by explants from a skin punch biopsy after informed consent [22,23,24] were grown in high-glucose Dulbecco’s modified Eagle’s medium (DMEM) supplemented with 10% (*v*/*v*) fetal bovine serum (FBS), 1% (*v*/*v*) L-glutamine, and 1% (*v*/*v*) penicillin/streptomycin at 37 °C in a humidified atmosphere of 5% CO_2_. For treatment conditions, cells were seeded in 96-well plates and grown for 24 h. The media were then removed, and cells were cultured at 37 °C in culture plates for 24 and 48 h in media containing different concentrations of lyophilized beer 

### 2.5. Cell Viability

Cell viability was assessed by the 3-(4,5-dimethylthiazol-2-yl)2,5-diphenyltetrazolium (MTT) assay [25] after 24 and 48 h of exposure of the cells and seeded in 96-well plates to the different concentrations of lyophilized beer samples. After the incubation, 150 µL DMEM and 15 µL of MTT (5 mg/mL) were added to each well. The plates were incubated for 3 h at 37 °C. The media were removed, and formazan crystals were dissolved in 150 µL isopropanol by gentle shaking. Absorbance was measured at 570 nm using a Victor 2030 multi-label reader (PerkinElmer, Waltham, MA, USA).

### 2.6. Determination of Reactive Oxygen Species (ROS)

The H_2_O_2_ levels were measured using the cell-permeant probe 2′-7′-dichlorodihydrofluorescindiacetate (H_2_DCFDA). Briefly, after 24 and 48 h of exposure to lyophilized beer samples, the media were changed and oxidative stress was induced by treatment with 50 µM tert-butyl hydroperoxide (t-BHP) (Sigma-Aldrich, B2633, St. Louis, MO, USA), as described in [26]. After 45 min of treatment, the cells were incubated with 10 µM H_2_DCFDA for 20 min at 37 °C in the dark. The cells were then washed and resuspended in 150 µL PBS. The H_2_O_2_-dependent oxidation of the fluorescent probe was measured (at 507 nm excitation and 530 nm emission wavelength) using the Victor 2030 Multilabel Reader (PerkinElmer, Waltham, MA, USA).

### 2.7. RNA Isolation Reverse Transcription and Quantitative PCR

The purification of total RNA from cells was carried out by using Aurum Total RNAMini Kit (Bio-Rad, Hercules, CA, USA) according to the manufacturer’s protocol. Complementary DNA (cDNA) was synthesized by reverse-transcription of total RNA using the iScriptcDNA Synthesis kit (Bio-Rad, Hercules, CA, USA), following the manufacturer’s instructions. Semi-quantitative determination of mRNA levels was performed by Real-Time Quantitative Reverse Transcription PCR (qRT-PCR) using SsoAdvanced Universal SYBRR Green Supermix (Bio-Rad, Hercules, CA, USA). Reactions were performed in a CFX96 Touch Real-Time PCR Detection System (Bio-Rad Laboratories, Hercules, CA, USA) in duplicate for each sample for three independent experiments. Relative quantification was performed using the comparative CT method (ΔΔCT). Quantitative normalization for each sample was performed by using glyceraldehyde-3-phosphate dehydrogenase (*GAPDH*) as an internal control. Validated primers for semi-qRT-PCR are provided in Table 2.

### 2.8. Statistical Analysis

Data are reported as mean ± standard error mean (SEM) of at least three independent experiments. Analysis of variance was carried out for all the data sets. One-way ANOVA with Tukey’s honestly significant test were performed. Powerful Statistical Analysis and Graphics Software for Windows 7 was used for all the statistical analyses. Effects were considered significant at *p* ≤ 0.05.

## 3. Results and Discussion

### 3.1. Effect of Antioxidant Compounds

**Figure 1:**
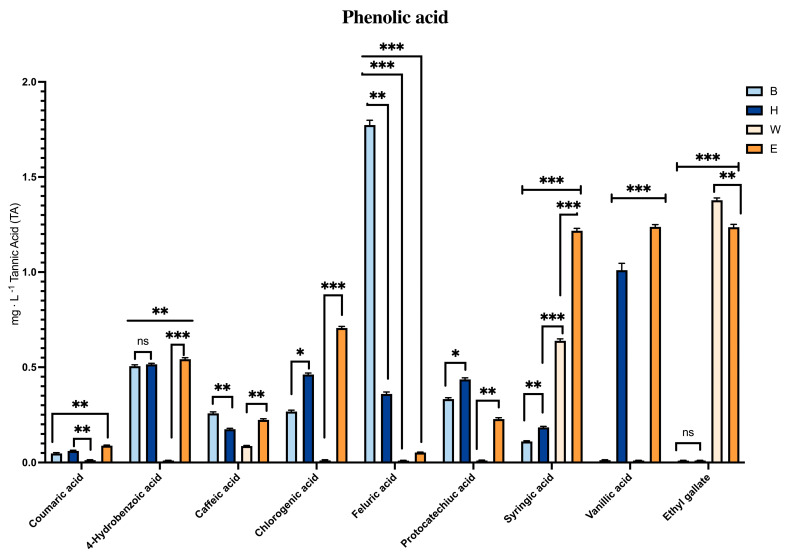
Phenolic acid content (mg · L^−1^ of tannic acid (TA)) in different craft beers. Data are the mean of 3 replications ± standard deviation. Statistical analyses were performed using Brown–Forsythe and Welch’s one-way analysis of variance. * *p* < 0.05, ** *p* < 0.01, *** *p* < 0.001, and not significant (ns). Asterisks indicate statistically significant differences. B, Blanche base beer; H, Heraclea (Blanche with the addition of bergamot juice extract, not filtered and not pasteurized); W, Weiss base beer; E, Elais (Weiss with the addition of olive extract, not filtered and not pasteurized).

As reported by Muscolo et al. [4], the Heraclea and Elais beers, both enriched with natural extracts, exhibited a lower protein content (0.2 g/100 mL) compared to the baseline Weiss and Blanche samples. This reduction in protein is of particular importance, as protein–polysaccharide interactions can lead to the formation of insoluble complexes that contribute to turbidity and negatively affect the physical stability of beer. The lower protein content observed in Heraclea and Elais may confer improved colloidal stability relative to the control beers. Total carbohydrate levels were slightly lower in the basal Weiss and Blanche than in the Heraclea and Elais beers but remained within the recommended range of 3.3–4.4 g/100 mL. Heraclea and Elais beers exhibited markedly higher levels of vitamin C—approximately 20-fold greater than those detected in the baseline Weiss and Blanche samples. Vitamin C is a potent antioxidant known to protect cellular components such as lipids, proteins, and nucleic acids from oxidative damage, while also playing a critical role in immune regulation, cell proliferation, and differentiation. Consistent with their elevated antioxidant content, Heraclea and Elais demonstrated significantly higher DPPH radical scavenging activity and total antioxidant capacity (TAC), indicating a stronger overall antioxidant potential. Regarding bioactive compounds, Elais showed the highest total polyphenol content, followed by Heraclea, with a similar trend observed for total flavonoids. The phenolic profiles of the beers analyzed in this study revealed distinct differences between the baseline formulations and those supplemented with bergamot and olive extracts, underscoring the impact of natural additive inclusion on the functional properties of the final product. Bergamot and olive are rich in phenolic compounds, including flavonoids and phenolic acids that when incorporated into beer enhance the beverage’s phenolic content. The bergamot addition contributed a flavonoid increase such as neoeriocitrin, naringin, and neohesperidin, which are known for their antioxidant properties. Similarly, the olive extracts introduced phenolics like syringic and ethyl gallate, compounds associated with various health benefits [4] (Figure 1).

Coumaric acid, although less representative than the other phenolic acids in all the beers, was present in the highest amount in Elais followed by Heraclea. Chlorogenic acid was also mostly present in Elais and Heraclea, syringic and ethyl gallate acids were significantly more abundant in Elais than in Heraclea and basal beers, while the protocatechuic acid was more concentrated in Heraclea than in the other beers. Additionally, p-coumaric acid, a phenolic compound found in various plant sources such as bergamot, has been associated with several health benefits. It is known for its strong antioxidant activity, which may help protect cells from oxidative stress [27]. p-Coumaric acid has been shown to possess anti-inflammatory properties, which may help reduce chronic inflammation linked to several health conditions, including cardiovascular diseases [28]. Research also suggests that p-coumaric acid may play a role in cancer prevention by inhibiting the growth of cancer cells and inducing apoptosis in certain types of cancer [29]. Studies have also suggested that p-coumaric acid may support cardiovascular health by reducing the risk of atherosclerosis and improving blood vessel function [30]. Chlorogenic acid, as reported in Kumar et al. [31], is considered an antioxidant, glycemic controller, anti-hypertensive, anti-inflammatory, antimicrobial, neuro-protective, and anti-obesity agent. It primarily activates the AMP-activated protein kinase, inhibits 3-hydroxy 3-methylglutaryl coenzyme-A reductase, and enhances the activity of carnitine palmitoyl transferase to control obesity. Ethyl gallate and syringic acid, both more expressed in Elais beer, can be considered markers of the olive extract addition to beer. In fact, syringic acid, as reported by Bartel et al. [32], is present in large amounts in olive fruit and the study assessed its pivotal effects on oxidative stress and inflammatory parameters. It is also effective on metabolic risk factors as well, including hyperglycemia, high blood pressure, and hyperlipidemia [32]. SA is one of the prominent polyphenolic compounds that may help address health issues related to civilization diseases. Ethyl gallate, which was identified by Muddu et al. [33] as inhibiting the hydrogen peroxide signaling, may instead represent a valid alternative class of vasopressors before use in septic shock. Osorio-Paz et al. [34] demonstrated that vanillic acid with well-attributed antioxidant, anti-inflammatory, and neuro-protective features may have beneficial health effects in extending health and lifespan. Protocatechuic acid was instead much more abundant in Heraclea, and it could be considered as the marker for the addition of bergamot juice extract to the basal Blanche beer. The effects of protocatechuic acid on health are well documented and evidenced by a wide range of pharmacological activities including antioxidant, anti-inflammatory, neuro-protective, antibacterial, antiviral, anticancer, anti-osteoporotic, analgesic, anti-ageing activities with consequent protection against metabolic syndrome and the preservation of liver, kidney, and reproductive functions [35]. The beers with the natural extracts added had a greater amount of polyphenols and single phenolic acids even if they exhibited significant differences from each other in the single phenolic acid concentrations.

**Figure 2:**
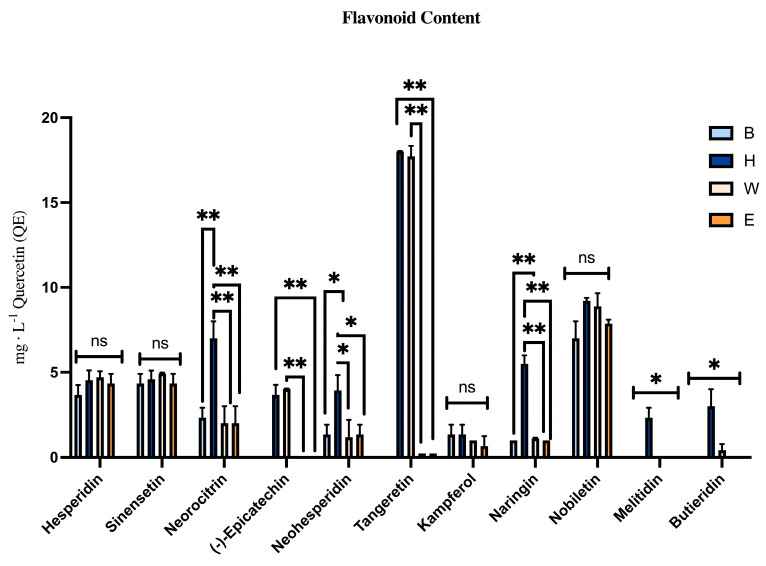
Flavonoids content (mg · L^−1^ Quercetin) in different craft beers. Data are the mean of 3 replications ± standard deviation. Statistical analyses were performed using Brown–Forsythe and Welch’s one-way analysis of variance. * *p* < 0.05, ** *p* < 0.01, and not significant (ns). Asterisks indicate statistically significant differences. B, Blanche base beer; H, Heraclea (Blanche with the addition of bergamot juice extract, not filtered and not pasteurized); W, Weiss base beer; E, Elais (Weiss with the addition of olive extract, not filtered and not pasteurized).

The profile of Heraclea was notably richer in both the variety and quantity of flavonoids compared to the other beers. Neoeriocitrin, naringin, and neohesperidin were highly expressed in Heraclea mainly because they are the bioactive molecules present in high concentrations in bergamot fruit (Figure 2).

These compounds exhibit a range of beneficial activities, such as antidiabetic, antiatherogenic, antidepressant, immunomodulatory, anticancer, anti-inflammatory, DNA protection, hypolipidemic, antioxidant, and cognitive-enhancing effects [36]. Melitidin and Brutieridin, present only in Heraclea, are unique to bergamot. These molecules are structurally similar to statins (drugs used to lower blood cholesterol and fatty acids), and several studies have suggested their involvement in inhibiting HMG-CoA reductase [37,38], leading to reduced cholesterol and fatty acids in human blood, contributing to their hypocholesterolemic properties. In short, the results showed significant differences between the beers enriched with natural extracts and the basal ones. Additionally, significant differences were found between the profiles of the enriched beers. When comparing Elais with Heraclea, a substantially lower total phenolic content was observed in Heraclea. Elais had a more complex phenolic profile compared to Heraclea due to the inclusion of olive extract, which is the richest in phenolic compounds. Conversely, Heraclea exhibited a superior qualitative and quantitative flavonoid profile compared to Elais, suggesting a different potentiality of the two beers in inducing health benefits for the possibility of activating/stimulating different metabolic pathways.

### 3.2. Effects of Lyophilized Beer Samples on Cell Viability

To assess the potential effects of the lyophilized beer, highly characterized primary human dermal fibroblasts isolated from a healthy subject were used. A cell viability test was used to monitor the viable cells with active metabolism after the exposure of cells in culture to the different beers at 24 and 48 h of treatments following the chemical reduction of 3-(4,5-dimethylthiazol-2-yl)-2,5-diphenyltet-razolium bromide (MTT) by mitochondrial reductases in living cells [26]

**Figure 3:**
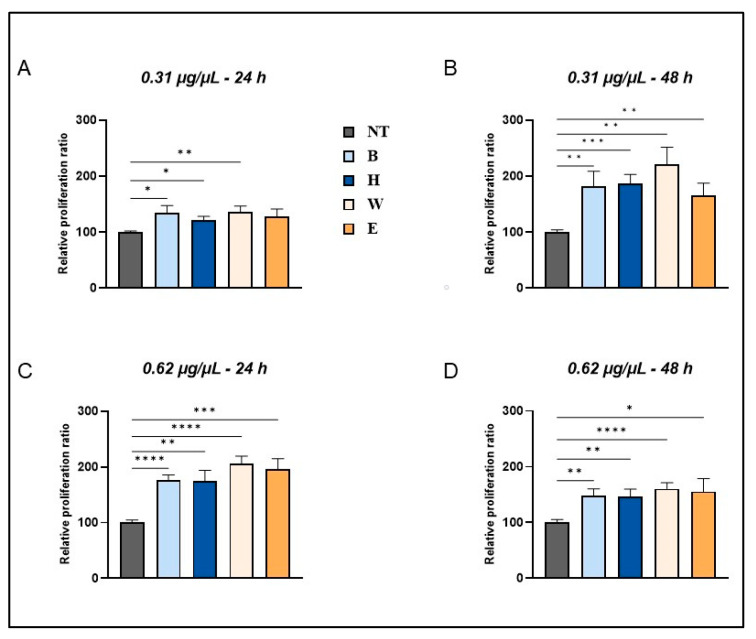
Enhanced cellular viability in beer-treated human fibroblasts. Cells were treated with 0.31 µg/µL (**A**,**B**) and 0.62 µg/µL (**C**,**D**) of different beer samples for 24 (**A**–**C**) and 48 h (**B**–**D**). Data mean ± SEM from at least three independent experiments under each condition are expressed as the percentage of vehicle-treated cells (NT). Statistical analyses were performed using Brown–Forsythe and Welch’s one-way analysis of variance. * *p* < 0.05, ** *p* < 0.01, *** *p* < 0.001, and **** *p* < 0.0001. B, Blanche basal beer; H, Heraclea (Blanche with the addition of bergamot juice extract, not filtered and not pasteurized); W, Weiss basal beer; E, Elais (Weiss with the addition of olive extract not filtered and not pasteurized).

As shown in Figure 3, treatment with the different beer samples resulted in a dose- and time-dependent response on cell viability. No toxic effects were observed in the presence of all the beer samples under different treatment conditions. Notably, a significant increase in cell proliferation was observed in cells treated with 0.31 µg/µL of all the lyophilized beer samples after 24 h of exposure, reaching 35% and 21% in the presence of Blanche and Heraclea, respectively, and 30% in the presence of Weiss and Elais, compared to untreated cells (Figure 3A). This effect persisted and significantly increased in cells treated with 0.31 µg/µL even at the longest incubation time (Figure 3B). Fibroblasts treated for 24 h with a double concentration (0.62 µg/µL) of beer samples showed an increase in viability of 70% in the presence of Blanche and Heraclea and 100% in the presence of Weiss and Elais (Figure 3C). At the longest incubation time (48 h), treatment with 0,62 µg/µL of the same beer samples elicited a lower response as compared to the 24 h treatment (Figure 3D).

As already shown by Ebadi and Fazeli [39], this response could be ascribed to the longer contact time of some phenolic acids with fibroblasts, combined with a higher concentration and exposure time. No significant differences in cell viability were observed in the presence of enriched beers compared to the respective basal beers.

### 3.3. Effects of Lyophilized Beer Samples on Reactive Oxygen Species Level

**Figure 4:**
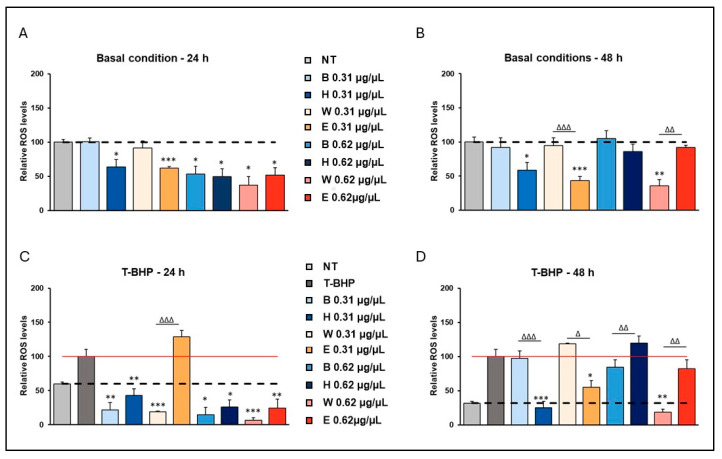
Reduced H_2_O_2_ levels in beer-treated human fibroblasts. Cells were treated with 0.31 µg/µL and 0.61 µg/µL of different beer samples for 24 and 48 h. ROS levels were measured by the DCF assay under basal conditions (**A**,**B**) and after exposure to the exogenous oxidative stress inducer t-BHP (**C**,**D**). Data mean ± SEM from at least three independent experiments under each condition are expressed as a percentage of vehicle-treated cells (dotted line) or tert-butyl hydroperoxide (t-BPH)-treated cells (red line). Statistical analyses were performed using Brown–Forsythe and Welch’s one-way analysis of variance. * *p* < 0.05, ** *p* < 0.01 and *** *p* < 0.001. Asterisks indicate statistically significant differences compared to vehicle or T-BPH-treated cells. Triangles indicate statistical significance (Brown–Forsythe and Welch’s one-way analysis of variance) as compared to the respective base beers. Δ *p* < 0.05, ΔΔ *p* < 0.01 and ΔΔΔ *p* < 0.001. NT, Not treated; t-BHP, tert-butyl hydroperoxide; B, Blanche base beer; H, Heraclea (Blanche with the addition of bergamot juice extract, not filtered and not pasteurized); W, Weiss base beer; E, Elais (Weiss with the addition of olive extract, not filtered and not pasteurized.

Recently, the potential anti-ageing benefits of polyphenols have attracted increasing scientific interest due to their ability to modulate cellular oxidative damage at 62 µg/µL. The effect of beer treatments on cellular ROS levels was then investigated to determine whether the improved vitality observed in the presence of lyophilized beers was related to their antioxidant activity. Specifically, the cells were treated with 0.31 and 0.62 µg/µL of beer samples for 24 and 48 h, as previously described. The basal ROS levels of cells treated with basal beers Blanche and Weiss (0.31 µg/µL) were not affected at either 24 (Figure 4A) or 48 h (Figure 4B). However, a significant decrease was observed in the presence of 0.31 µg/µL Heraclea (Blanche beer with bergamot juice extract) and 0.31 µg/µL Elais (Weiss with olive extract) at both incubation times (Figure 4A,B). Elais and Heraclea have the highest levels of total phenolic acids, compared to the basal beers Blanche and Weiss, which could exert a powerful antioxidant action to protect against reactive oxygen species (ROS)/cellular oxidative stress [40]. In the presence of a higher concentration of beer (0.62 µg/µL), a decrease in basal ROS levels was observed after 24 h of treatment for all beer samples compared to untreated cells (Figure 4A). Nevertheless, a decrease in basal ROS levels after 48 h of treatment was observed only in the Weiss beer-treated cells (Figure 4B). The lack of the antioxidant effect of the other beer samples could be ascribed to the possibility that some antioxidants can exhibit pro-oxidant behavior under certain conditions in the biological systems [41,42,43]. Pro-oxidant behavior allows cells to respond quickly to oxidative stress by activating various protective mechanisms [39]. To assess the effect of beer exposure under an oxidative stress environment, fibroblasts were pre-treated with the beer samples, as described previously, and then incubated in the presence of t-BPH for 45 min, as described in Matrella et al. [26]. t-BHP cell treatments induce a significant increase in intracellular ROS and mitochondrial depolarization and reduced mitochondrial viability [39]. As expected, a significant increase in cellular ROS levels was measured in the t-BPH-treated fibroblasts, at 24 (Figure 4C) and 48 h (Figure 4D). A significant reduction in t-BPH-induced ROS levels was observed after pre-treatment with both low and high concentrations of all beer samples over an incubation period of 24 h, compared to t-BPH-treated cells, except for pre-treatment with low concentrations of Elais, which resulted in increased ROS levels comparable to that induced by t-BPH treatment (Figure 4C). After 48 h of pre-treatment, a significant reduction in t-BPH-induced ROS level was observed with 0.31 µg/µL Heraclea and Elais beer (Figure 4D). It should be noted that this significant reduction in the ROS level induced by pre-treatment with the enriched beers Heraclea and Elais is also relevant compared to the pre-treatment with the basal beers Blanche and Weiss, respectively (Figure 4D).

### 3.4. Effects of Lyophilized Beer Samples on RNA Levels of Antioxidant Enzymes

Given these results, we hypothesized that the antioxidant function of beers may be due not only to a direct action of the beer components, but also to the activation of cellular responses that regulate gene expression levels of antioxidant enzymes. The first line of defense in the cells is antioxidant enzymes such as superoxide dismutases (SODs), catalase (CAT), and glutathione peroxidase (GPxs) involved in the dismutation of superoxide radicals and hydrogen peroxide [44]. The impact of the beer treatment was assessed by comparing changes in the transcript levels of genes encoding antioxidant enzymes through real-time quantitative polymerase chain reaction (qPCR) analysis in fibroblasts treated for 24 h with 0.31 µg/µL and 0.62 µg/µL of the distinct beer samples. Under basal conditions, the scavenger gene expression was not affected by the beer sample treatments, at concentrations of 0.31 µg/µL and 0.62 µg/µL (Figures S1 and S2, Supplementaty Materials). This evidence indicates that the observed reduction in ROS levels following exposure to 0.31 μg/μL of the Heraclea and Elais and 0.62 µg/µL of the basal and enriched beer samples may be attributed to the intrinsic antioxidant properties of the polyphenols and phytochemicals present in the beer extracts, rather than the activation of antioxidant enzymatic systems for ROS removal.

**Figure 5:**
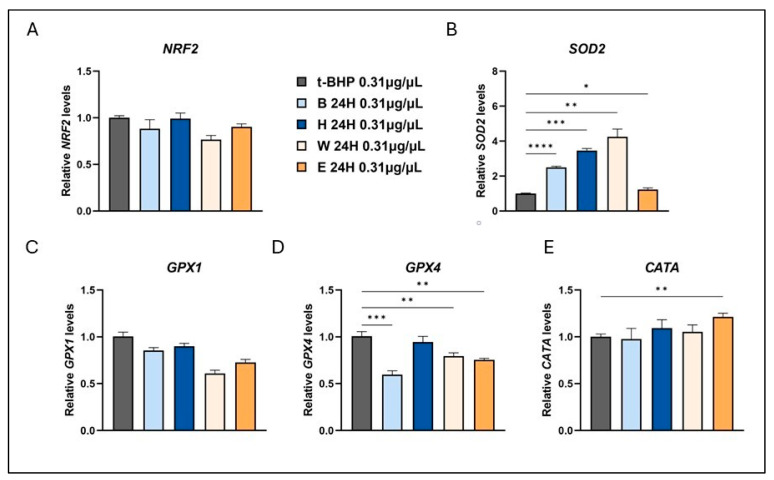
Modulation of antioxidant enzyme expression in beer-treated human fibroblasts. Cells were treated with 0.31 µg/µL of different beer samples for 24 h. Transcript levels of NRF2 (**A**), SOD2 (**B**), GPX1 (**C**), GPX4 (**D**), and CATA (**E**) were measured after exposure to the exogenous oxidative stress inducer t-BHP. Relative mRNA levels were evaluated by qRT-PCR and normalized to the housekeeping gene GAPDH. Data expressed as fold-change mRNA expression levels in beer-treated cells, compared to t-BPH-treated cells, are means ± SEM of two replicates from three independent experiments. Statistical analyses were performed using Brown–Forsythe and Welch’s one-way analysis of variance. * *p* < 0.05, ** *p* < 0.01, *** *p* < 0.001, **** *p* < 0.0001. Asterisks indicate statistically significant differences compared to T-BPH-treated cells. B, Blanche base beer; H, Heraclea (Blanche with the addition of bergamot juice extract, not filtered and not pasteurized); W, Weiss base beer; E, Elais (Weiss with the addition of olive extract, not filtered and not pasteurized).

To assess the gene expression response to an oxidative stress condition, the t-BHP was employed as the inducing agent, as previously described [26]. Following pre-treatment with low concentrations (0.31 µg/µL) of beer samples, a significant fold increase in SOD2 expression was observed in all treated groups, except for Elais, compared to the t-BPH-treated control cells (Figure 5B). The upregulation of SOD2 gene expression, one of the key antioxidant enzymes, in response to oxidative stress induced by t-BHP reflects an adaptive cellular mechanism against oxidative damage, which differs from the outcomes observed under basal conditions. SOD2 is a mitochondrial protein which binds to superoxide byproducts of oxidative phosphorylation and converts them to hydrogen peroxide. Additionally, a significant reduction in GPX4 expression levels was detected in the cells pre-treated with Blanche beer (Figure 5D).

**Figure 6:**
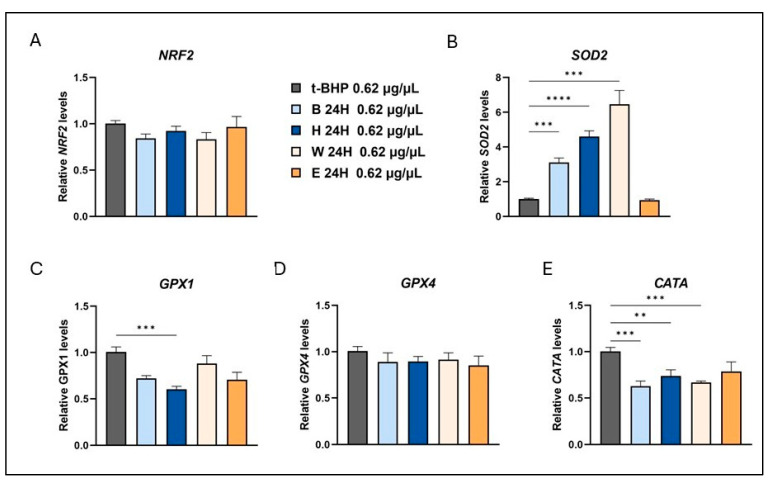
Modulation of antioxidant enzyme expression by beer samples in t-BHP-treated cells. Cells were treated with 0.62 µg/µL of different beer samples for 24 h. Transcript levels of NRF2 (**A**), SOD2 (**B**), GPX1 (**C**), GPX4 (**D**), and CATA (**E**) were measured after exposure to the exogenous oxidative stress inducer t-BHP. Relative mRNA levels were evaluated by qRT-PCR and normalized to the housekeeping gene GAPDH. Data, expressed as fold-change mRNA expression levels in beer-treated cells, compared to t-BPH-treated cells, are means ± SEM of two replicates from three independent experiments. Statistical analyses were performed using Brown–Forsythe and Welch’s one-way analysis of variance. ** *p* < 0.01, *** *p* < 0.001, and **** *p* < 0.0001. Asterisks indicate statistically significant differences compared to T-BPH-treated cells. B, Blanche base beer; H, Heraclea (Blanche with the addition of bergamot juice extract, not filtered and not pasteurized); W, Weiss base beer; E, Elais (Weiss with the addition of olive extract, not filtered and not pasteurized).

Consistent with the effects observed at a concentration of 0.31 μg/μL, treatment with a higher concentration (0.62 μg/μL) of the beer samples resulted in a significant fold increase in SOD2 gene expression for all beer samples, except for Elais, in comparison to t-BHP-treated cells (Figure 6B). The increase in SOD2 expression without the simultaneous increase in catalase and glutathione peroxidase could lead to an increase in hydrogen peroxide contributing to cell signaling [45]. Several studies have shown that increased intracellular levels of reactive oxygen species can stimulate AMPK activity [46,47]. AMPK is the primary sensor of cellular energy which regulates diverse metabolic and physiological processes, stress responses, and cell growth [48].

**Figure 7:**
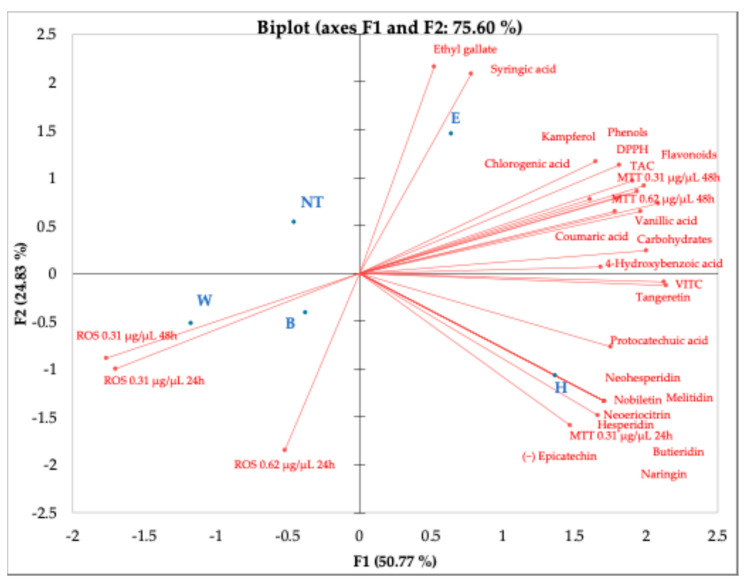
Principal component analysis (PCA) shows how the variables (total phenols, total flavonoids, phenolic acids, flavonoids, and vitamins) contained in the different beers relate to each other, to reactive oxygen species, and to cell viability (MTT) at two different concentration (0.31 and 0.62 µg/µL) and exposure time (24 and 48 h). B, Blanche base beer; H, Heraclea (Blanche with the addition of bergamot juice extract, not filtered, and not pasteurized); W, Weiss base beer; E, Elais (Weiss with the addition of olive extract, not filtered, and not pasteurized).

**Figure 8:**
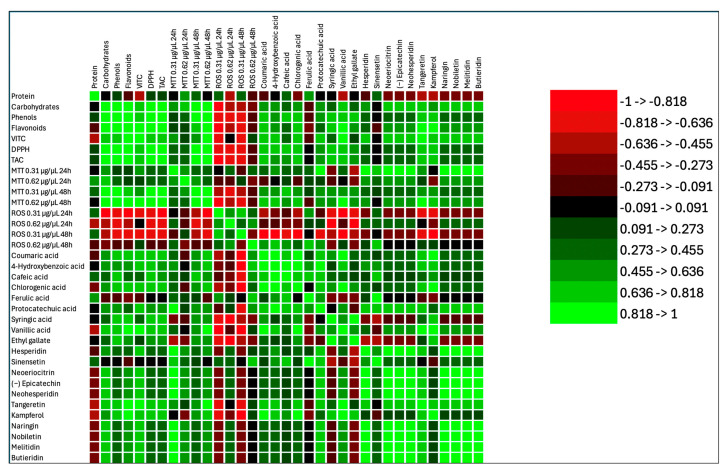
Correlation matrix (Pearson (n − 1)) of different compounds (total phenols, total flavonoids, phenolic acids, flavonoids, and vitamins) contained in the different beers relate to each other, to reactive oxygen species (ROS), and to cell viability (MTT) at two different concentration. Green color and its shades, in the correlation matrix, indicate a positive correlation, signifying that the variables move in the same direction. On the other hand, the red color and its gradients represent an inverse correlation, suggesting that the variables move in the opposite direction.

Data from PCA analysis evidenced that Elais and Heraclea beers behave differently. Elais correlated with antioxidant compounds (phenolic acids, DPPH, TAC, carbohydrates, and MTT 0.31 and 0.62 at 48 h exposure). Conversely, Heraclea correlated with flavonoids, vitamin C, and MTT 0.31 at 24 h exposure. Basal beers Weiss and Blanche correlated only with ROS both at 24 and 48 h exposure (Figure 7). These findings are supported by the correlation heatmap that provides valuable insights into the relationships among biochemical compounds, antioxidant activity, cytotoxicity, and oxidative stress parameters (Figure 8). A strong positive correlation (light green) was observed between total phenols, flavonoids, and antioxidant activity (DPPH, TAC), indicating that these compounds significantly contribute to the overall antioxidant potential. Furthermore, several phenolic acids (e.g., ferulic acid, chlorogenic acid, epicatechin) showed a highly significant correlation with TAC and DPPH, suggesting their essential role in free radical scavenging. Conversely, ROS levels exhibited a negative correlation (red) with most phenolic compounds, implying that these bioactive molecules effectively reduce oxidative stress. In particular, flavonoids such as hesperidin, neohesperidin, and naringin displayed strong inverse correlations with ROS, reinforcing their protective role against oxidative damage. Regarding cytotoxicity (MTT assay), higher concentrations and longer exposure times (MTT 0.62 µg/µL at 48 h) showed negative correlations with some flavonoids, suggesting potential cytotoxic effects at prolonged exposure. Interestingly, some phenolic acids, including p-coumaric acid and protocatechuic acid, showed mixed correlations with ROS and MTT, suggesting a concentration-dependent or dual role in modulating oxidative stress. Additionally, flavonoids exhibited strong intercorrelations, suggesting possible synergistic effects in modulating antioxidant activity and cellular responses. Overall, these findings highlighted the significant role of phenols and flavonoids in antioxidant defense, cytotoxicity modulation, and oxidative stress regulation, with potential implications for therapeutic applications.

## 4. Conclusions

This study provides the first integrated biochemical and molecular characterization of craft beers enriched with bergamot (Heraclea) and olive (Elais) extracts, revealing their distinct and composition-specific impacts on antioxidant defense systems. Through a comprehensive analytical approach combining UHPLC-based polyphenolic profiling, ROS quantification, cell viability assays, and qPCR gene expression analysis, we demonstrated that the bioactive matrix of these functional beers exerts differential modulatory effects on cellular redox homeostasis. Heraclea beer, enriched with bergamot juice, displayed a superior flavonoid composition, including neohesperidin, naringin, and brutieridin, which strongly correlated with reduced intracellular ROS and enhanced fibroblast viability. These effects were more pronounced at 0.31 µg/µL and 24 h, indicating a rapid and efficient antioxidant response likely mediated by high-affinity polyphenol–cellular interactions. In contrast, the Elais beer, containing olive extract, exhibited a more complex phenolic acid profile—dominated by ethyl gallate, syringic acid, and chlorogenic acid—associated with an elevated total antioxidant capacity and polyphenol-driven ROS scavenging.

Functionally, both enriched beers upregulated SOD2 expression under oxidative stress, with Heraclea showing a more consistent transcriptional activation across experimental conditions. PCA and correlation analysis confirmed the functional divergence of the two formulations: Heraclea correlated with flavonoid abundance and early cytoprotection, while Elais aligned with prolonged antioxidant activity and phenolic acid content. These findings reveal two mechanistically distinct but complementary antioxidant strategies embedded in the two formulations. Interestingly, a loss of antioxidant efficacy or potential pro-oxidant shift was observed at higher concentrations (0.62 µg/µL) and longer exposure (48 h), consistent with the hormetic behavior of polyphenols. This underscores the importance of dose optimization in designing functional foods for maximal health benefit without triggering cytotoxic thresholds. In conclusion, this study substantiates the feasibility of designing phenolic-rich craft beers with targeted biofunctional properties. By linking the chemical composition to cellular and genetic outcomes, we provide novel insights into how specific botanical matrices can tailor antioxidant responses. These data support the positioning of enriched beers not merely as enhanced sensory products but as functional beverages with defined bioactivity. Further translational studies, including in vivo validation and pharmacokinetic profiling, will be crucial to establish clinical relevance and inform regulatory guidelines for functional alcoholic beverages.

## Figures and Tables

**Table 1:** Retention time, instrumental recovery, and percentage relative standard deviation of polyphenols.

Compounds	Rt	±SD	RSD%	Recovery (%)
Coumaric acid	12.396	0.008	0.496	102.1
4-Hydroxybenzoic acid	4.060	0.014	1.310	103.8
Caffeic acid	7.751	0.011	0.601	102.0
Ethyl gallate	9.836	0.020	1.396	102.7
Ferulic acid	11.323	0.007	0.258	101.9
Kampferol	18.551	0.014	0.767	100.8
Protocatechuic acid	2.157	0.009	0.769	102.3
Syringic acid	8.996	0.008	0.283	97.3
Vanillic acid	7.344	0.015	1.172	95.9
Chlorogenic acid	8.637	0.015	0.837	95.9
Naringin	14.125	0.010	0.358	93.2
Hesperidin	14.531	0.007	0.321	95.8
Sinensetin	21.488	0.006	0.385	108.2
Neoeriocitrin	12.986	0.011	0.772	95.1
(-)Epicatechin	9.880	0.013	0.868	93.9
Neohesperidin	14.925	0.042	0.604	100.8
Tangeretin	23.290	0.019	0.964	104.4
Melitidin	11.422	0.009	0.655	102.8
Nobiletin	22.432	0.006	0.077	99.7
Butieridin	11.611	0.011	0.101	99.6

**Table 2:** Oligonucleotide sequences for real-time PCR.

Gene	Forward Primer Sequence	Reverse Primer Sequence
NRF2	5′-AGCCCAGCACATCCAGTCA-3′	5′-TGTGGGCAACCTGGGAGTAG-3′
SOD2	5′-CTGGACAAACCTCAGCCCT-3′	5′-CTGATTTGGACAAGCAGCAA-3′
GPX1	5′-AGAACGCCAAGAAGCAGAAGA-3′	5′-CATGAAGTTGGGCTCGAACC-3′
GPX4	5′-AACTACACTCAGCTGCTGC-3′	5′-GCAGGTCTTCTCTCATCACC-3′
CATA	5′-TGGAAAGAAGACTCCCATCG-3′	5′-CCAGAGATCCCAGACCATGT-3′
GAPDH	5′-CAACTTTGGTATCGTGGAAGGAC-3′	5′-ACAGTCTTCTGGGTGGCAGTG-3′

## Data Availability

The original contributions presented in this study are included in the article; further inquiries can be directed to the corresponding author.

## Supplementary Materials

The following are available online at https://www.mdpi.com/article/10.3390/foods14122012/s1, Figure S1: Effects of lyophilized beer samples on RNA levels of antioxidant enzymes under basal conditions, Figure S2. Effects of lyophilized beer samples on RNA levels of antioxidant enzymes under basal conditions.
