# Supplementary material for: Bergamot and Olive Extracts as Beer Ingredients: Impact on Cell Viability, Reactive Oxygen Species, and RNA Expression of Antioxidant Enzymes
Source: Foods. 2025 Jun 6;14(12):2012. doi: 10.3390/foods14122012 (PMC12191999; doi:10.3390/foods14122012)
Supplement: Supplementary file 1 [file foods-14-02012-s001.zip › foods-3570404-supplementary.pdf]

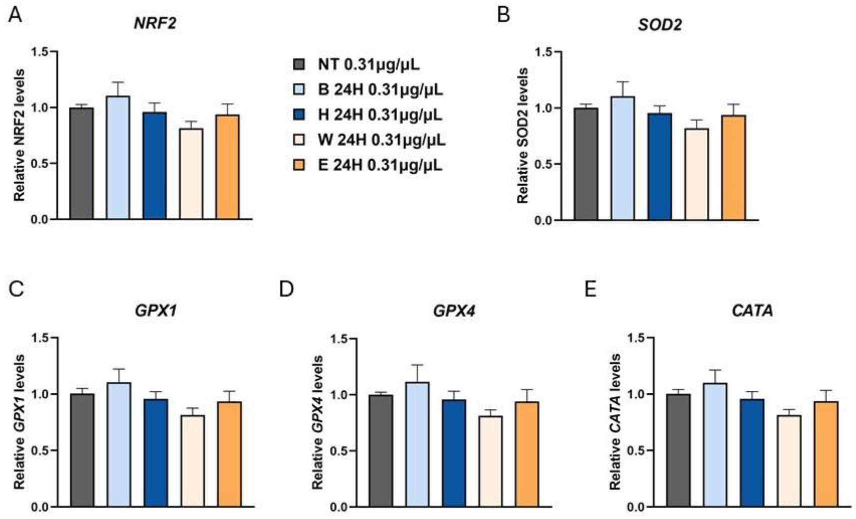

Figure S1. Effects of lyophilized beer samples on RNA levels of antioxidant enzymes under basal conditions. Cells were treated with 0.31 µg/µL of different lyophilised beer samples for 24 h. Transcript levels of *NRF2* **(A)**, *SOD2* **(B)**, *GPX1* **(C)**, *GPX4* **(D)** and *CATA* **(E)** were measured under basal conditions. Relative mRNA levels were evaluated by qRT-PCR and normalized to the housekeeping gene *GAPDH*. Data, expressed as fold-change mRNA expression levels in beer-treated cells, compared to untreated cells (NT), are means±SEM of two replicates from three independent experiments. Statistical analyses were performed using Brown-Forsythe and Welch's one-way analysis of variance. * $p < 0.05$, ** $p < 0.01$ and *** $p < 0.001$. Asterisks indicate statistically significant differences compared to vehicle-treated cells.

B, Blanche base beer; **H**, Heraclea (Blanche with the addition of bergamot juice extract, not filtered and not pasteurised); **W**, Weiss base beer; **E**, Elais (Weiss with the addition of olive extract, not filtered and not pasteurised)

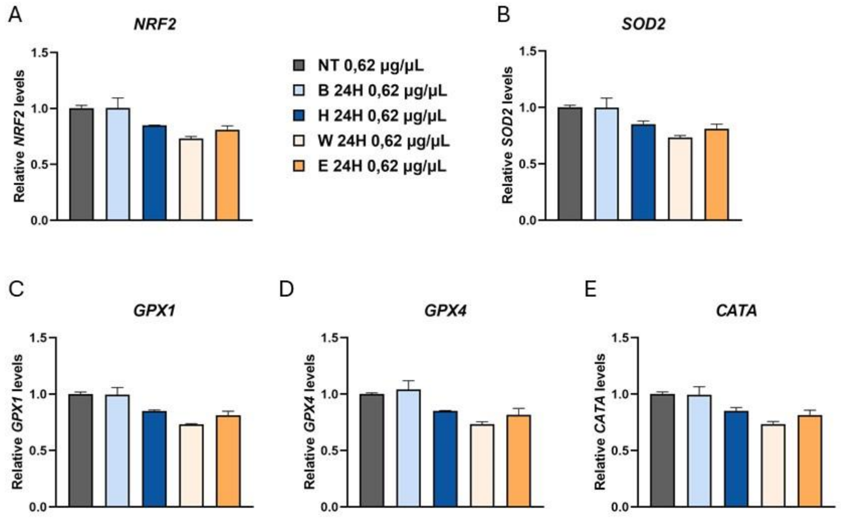

Figure S2. Effects of lyophilized beer samples on RNA levels of antioxidant enzymes under basal conditions. Cells were treated with 0.62 µg/µL of different lyophilised beer samples for 24 h. Transcript levels of *NRF2* **(A)**, *SOD2* **(B)**, *GPX1* **(C)**, *GPX4* **(D)** and *CATA* **(E)** were measured under basal conditions. Relative mRNA levels were evaluated by qRT-PCR and normalized to the housekeeping gene *GAPDH*. Data, expressed as fold-change mRNA expression levels in beer-treated cells, compared to untreated cells (NT), are means±SEM of two replicates from three independent experiments. Statistical analyses were performed using Brown-Forsythe and Welch's one-way analysis of variance. * $p < 0.05$, ** $p < 0.01$ and *** $p < 0.001$. Asterisks indicate statistically significant differences compared to vehicle treated cells.

B, Blanche base beer; **H**, Heraclea (Blanche with the addition of bergamot juice extract, not filtered and not pasteurised); **W**, Weiss base beer; **E**, Elais (Weiss with the addition of olive extract, not filtered and not pasteurised)
